# Bridging healthcare disparities: a systematic review of healthcare access for disabled individuals in rural and urban areas

**DOI:** 10.3389/frhs.2025.1695320

**Published:** 2025-11-18

**Authors:** Amer Mesmar, Godfrey Mbaabu Limungi, Mohammed Elmadani, Klara Simon, Osama Hamad, Livia Tóth, Eva Horvath, Orsolya Mate

**Affiliations:** 1 Doctoral School of Health Sciences, Faculty of Health Sciences, University of Pécs, Pécs, Hungary; 2 Faculty of Dentistry, University of Jordan, Amman, Jordan; 3 Institute of Emergency Care, Pedagogy of Health and Nursing Sciences, Faculty of Health Sciences, University of Pécs, Pécs, Hungary

**Keywords:** healthcare access, disability, health disparities, rural health, urban health, telemedicine, healthcare barriers, equity in healthcare

## Abstract

**Objective:**

This review will examine existing research to compare the differences in healthcare access for people with disabilities in rural vs. urban areas. The goal is to identify common obstacles and helpful factors that affect their ability to get healthcare, which can inform the creation of specific programs to close these gaps.

**Methods:**

This systematic review was pre-registered with PROSPERO (Registration No. CRD42025648258). A comprehensive search was conducted across databases including PubMed, Scopus, Web of Science, and the Cochrane Library, for peer-reviewed articles published between January 1, 2010, and December 31, 2024. Studies were included if they addressed healthcare access for disabled individuals and made comparisons between rural and urban settings. Data extraction was performed using standardized forms, and quality assessment was conducted using the Mixed Methods Appraisal Tool (MMAT). Data synthesis involved a narrative synthesis and thematic analysis to identify key barriers and facilitators to healthcare access in rural and urban areas. The reporting of this review follows the PRISMA guidelines.

**Results:**

Eight studies from Peru, China, the United States, Mozambique, and South Africa were included in the final review. A clear distinction emerged between the barriers to healthcare access in rural and urban areas. Rural settings were defined by infrastructure-related challenges, such as transportation difficulties, a lower number of healthcare facilities, and limited provider availability. Meanwhile, urban areas presented different barriers, including overcrowded facilities and extended wait times. Both settings struggled with socioeconomic disparities, but the specific barriers and facilitators varied. In rural areas, telemedicine and mobile clinics were identified as key facilitators, while in urban areas, specialized healthcare services and better public transportation were the most helpful in bridging access gaps.

**Conclusion:**

This systematic review confirms that disabled individuals face significant, yet distinct, healthcare access disparities depending on their location. In rural areas, the primary barriers are transportation and a lack of facilities, which necessitates the development of community-specific solutions such as mobile clinics and expanded telemedicine. In urban settings, access is hindered by system overcrowding and socioeconomic divides, calling for interventions that improve public transportation access and address systemic inequalities. Ultimately, addressing these disparities requires a dual approach: empowering rural communities with technological and logistical support while simultaneously optimizing urban healthcare systems to be more accessible and equitable.

**Systematic Review Registration:**

https://www.crd.york.ac.uk/PROSPERO/view/CRD42025648258, PROSPERO CRD42025648258.

## Introduction

Access to healthcare is a pressing global concern, particularly for individuals with disabilities, who constitute approximately 15% of the world's population (1). A significant majority of these individuals, around 80%, reside in low- and middle-income countries (2), where healthcare infrastructure and resources are often limited (3). These global disparities are exacerbated by persistent, widespread barriers such as inadequate transportation, physical accessibility challenges within facilities, and socioeconomic factors like a lack of insurance and high unemployment rates (4).

While these barriers are universal, their impact is shaped by geographic location. In rural areas, the primary challenges for disabled individuals often stem from a lack of infrastructure, including greater travel distances to facilities, limited availability of specialized care, and a shortage of healthcare personnel. Conversely, urban settings, despite offering a greater number of facilities, present their own set of barriers, such as system overcrowding, long wait times, and discriminatory attitudes. For disabled individuals, these issues are compounded by the need for tailored accommodations that are frequently unavailable or underdeveloped.

Adults with intellectual disabilities are particularly affected by these challenges, with the intersection of disability and rural living placing them at a distinct disadvantage (5). While existing research highlights general healthcare disparities between disabled and non-disabled populations, a notable gap remains in understanding the specific differences within the disabled community across various geographic settings (6). This systematic review aims to address this gap by investigating and comparing the distinct barriers and facilitators to healthcare access for disabled individuals in rural and urban environments, providing insights for developing tailored, context-specific interventions.

## Objectives

The main goal of this systematic review is to investigate and compare the differences in healthcare access for people with disabilities in rural vs. urban areas. We will achieve this by focusing on four key objectives:

- **Assessing Disparities:** We'll evaluate the full scope of healthcare access disparities, identifying the key differences in access and outcomes for disabled individuals based on their location.
- **Pinpointing Barriers:** We'll pinpoint the specific obstacles people face, from environmental and socioeconomic challenges to issues with infrastructure, in both rural and urban settings.
- **Highlighting Solutions:** We'll identify effective strategies and interventions that have successfully improved healthcare access, paying close attention to what works best in different geographic contexts.
- **Creating Recommendations:** Finally, we'll develop evidence-based recommendations for policymakers, healthcare providers, and community organizations. These recommendations will focus on practical, tailored solutions to address the unique challenges of each setting.

## Methodology

### Research question

The primary research question guiding this review is: What are the barriers and facilitators to healthcare access for disabled individuals in rural vs. urban areas?

### Protocol and registration

This systematic review was conducted in accordance with a pre-registered protocol. The protocol was registered in the International Prospective Register for Systematic Reviews (PROSPERO) on **February 5th, 2025**, with the registration number **CRD42025648258**. The review adheres to the standards outlined in the Preferred Reporting Items for Systematic Reviews and Meta-Analyses (PRISMA) statement, which was used as a guide for reporting all stages of the review process.

### Eligibility criteria

The review included all studies employing quantitative, qualitative, or mixed methods designs. To be included, studies had to be published in English in a peer-reviewed journal and had to focus on healthcare access for disabled individuals while also comparing or contrasting findings between rural and urban settings. Studies were excluded if they were discussion papers, dissertations, theses, commentaries, editorials, systematic reviews, scoping reviews, meta-analyses, or literature reviews. Dissertations and theses were excluded due to the potential for limited peer review, which may impact the reliability of their findings. Studies with low-quality evidence, such as case reports or case series, or those with insufficient data or an unclear methodology were also excluded.

### Databases and search strategies

Two research team members independently conducted a comprehensive literature search across six databases: PubMed, Scopus, CINAHL, Web of Science, Medline, and Embase. The search was conducted between **January 1, 2010, and December 31, 2024**, ensuring a focus on recent literature.

The search strategy included a combination of keywords and MeSH terms related to:

- **Disabled individuals:** (“disabled individuals” OR “people with disabilities” OR “disability”)
- **Healthcare access:** (“healthcare access” OR “health care access” OR “health disparities” OR “healthcare utilization”)
- **Geographic setting:** (“rural areas” OR “urban areas” OR “rural-urban”)

The full search strategy was adapted for each database's specific syntax to ensure maximum retrieval of relevant articles.

### Selection of sources of evidence

After retrieving articles from the databases, the search results obtained by both reviewers were uploaded to the Rayyan software, where duplicate records were automatically identified and removed using the platform's built-in duplicate detection feature. The unified list of unique studies was then used to facilitate the screening process. Two reviewers independently screened all articles by title and abstract to determine if they met the eligibility criteria. The process was blinded to prevent bias. Any disagreements between the two reviewers were resolved by a third reviewer. The full texts of all articles that passed the initial screening were then retrieved and independently evaluated for final inclusion. Discrepancies were again resolved through discussion and consensus among the three reviewers. The selection process is documented visually using a PRISMA flow diagram.

### Quality appraisal

An evaluation of the quality of individual studies was performed using the Mixed Methods Appraisal Tool (MMAT), which is an effective tool for assessing the quality of quantitative, qualitative, and mixed-methods research. MMAT was selected due to its unique flexibility in evaluating studies across multiple methodologies. The appraisal process considered various dimensions of bias, including selection bias, measurement bias, and reporting bias. While MMAT provided a comprehensive framework, some challenges arose during the evaluation, particularly when interpreting the risk of bias in studies with insufficient reporting. To address these challenges, a third reviewer was involved to resolve any discrepancies through discussion, ensuring consistency in the evaluations. Each study was categorized into one of three levels of bias: “low risk of bias”, “some concern of bias”, and “high level of bias”. Studies with a sufficient sample size, a well-elaborated methodology, and clear reporting were categorized as “low risk of bias”. Those that had some methodological limitations, such as a possible risk for self-reporting bias, were categorized as “some concern of bias”. Studies with insufficient sample size, unelaborated methodology, or a high likelihood of reporting bias were categorized as “high level of bias”. To ensure the accuracy of the evaluation, two research team members independently assessed the risk of bias and quality of each study. In case of disparity, they consulted the third research team member to reconcile any differences.

### Data charting process

This systematic review used a data extraction table developed by the principal researcher to systematically extract data from the included studies. The data charting tool was pilot-tested and refined by the research team to ensure it could reliably capture all intended data. Extracted data included study characteristics (author, year of publication, study design, and country), population details (age, type of disability), the specific setting of the study (rural or urban), key findings related to barriers and facilitators, and any reported outcomes. To ensure reliability, two reviewers independently extracted data from the included studies. Any discrepancies that arose during this process were resolved through discussion to reach a final consensus.

### Data synthesis and analysis

Data were synthesized qualitatively to describe and compare the findings from the included studies. A narrative synthesis was conducted to summarize the barriers and facilitators to healthcare access identified in rural and urban settings and to highlight common themes across the literature. A thematic analysis was also used to identify, analyze, and report patterns within the data. This involved an inductive coding approach, where themes related to barriers and facilitators emerged directly from the extracted data. To ensure the reliability and validity of this analysis, two reviewers independently coded the data before discussing and reaching a final consensus on the thematic framework. The final synthesis was cross-checked to ensure consistency and minimize potential bias.

## Results

This systematic review aimed to identify and analyze the key barriers and facilitators to healthcare access for people with disabilities in rural and urban areas. The initial search across all selected databases yielded a total of 1,400 potential articles. After removing 398 duplicate publications, 1,002 articles were selected for consideration based on their titles and abstracts. A majority of the included studies were published in the last decade, with the earliest publication dating back to 2011. A thorough title and abstract screening resulted in 970 publications being excluded.

**Figure 1:**
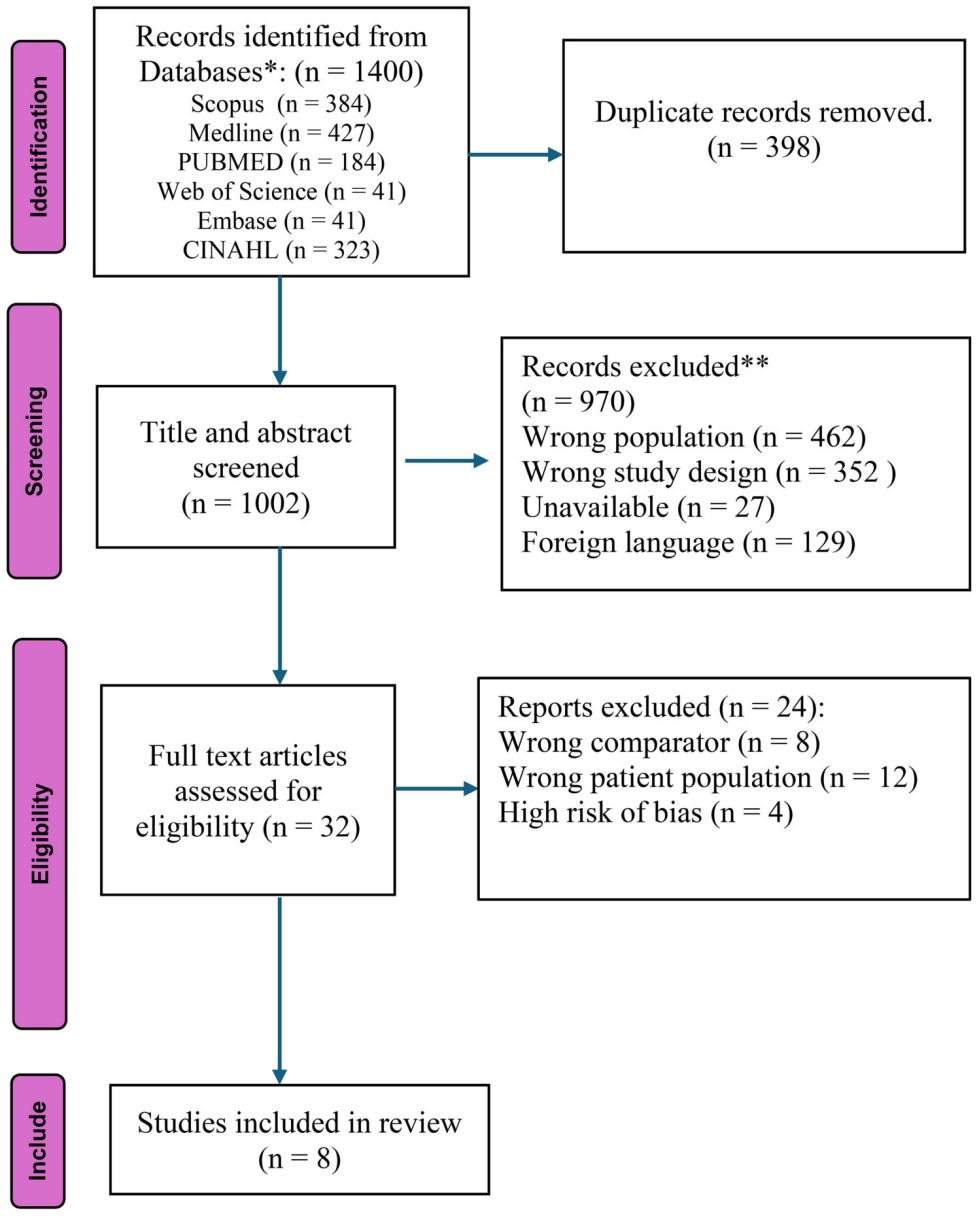
PRISMA flow diagram of study selection.

Of the 32 studies selected for full-text review, 24 were excluded after detailed assessment based on the predefined inclusion and exclusion criteria. Eight studies were excluded due to an inappropriate comparator, where the studies compared populations that did not align with the review objectives. Twelve studies were excluded for focusing on an ineligible patient population, such as individuals without documented disabilities or those residing exclusively in institutional care settings rather than community or primary healthcare contexts. Four additional studies were excluded due to a high risk of bias, primarily related to unclear sampling procedures, insufficient methodological transparency, or inadequate reporting of outcomes. Consequently, eight studies met all eligibility criteria and were included in the final synthesis for data extraction and thematic analysis. The characteristics of these included studies are summarized in Table 1, and the full study selection process is detailed in the PRISMA flowchart (Figure 1).

**Table 1:** Characteristics of the included studies, detailing author, year, country, setting, population, methodology.

Study	Type of study	Participants	Country
Moscoso-Porras et al. (3)	Quantitative cross-sectional	The study included 37,524 participants (57.5% female, mean age 66.5 years), with 20,663 identified as having a physical disability, and noted lower education and socioeconomic status among rural residents compared to their urban counterparts.	Peru
Zhao and Wang (6)	Quantitative cross-sectional	29,769 PWD, a representative sample from the Second China National Sample Survey on Disability.	China
Gimm and Ipsen (7)	Quantitative Cross-Sectional	Data from Wave 2 of the National Survey on Health and Disability (NSHD)	United States
Pinto and Muhache (2)	Qualitative Cross-Sectional study	34 people with disabilities	Mozambique
Zhang et al. (4)	Longitudinal study	26,604 adults aged 65 and older from four waves of the CLHLS (2005, 2008/2009, 2011/2012, 2014)	China
Hamilton et al. (8)	Qualitative (Focus groups and interviews)	53 participants with physical, cognitive, and mental disabilities from 12 counties in Mid-Michigan	USA
Grut et al. (9)	Qualitative study using in-depth and semi-structured interviews.	24 individuals with disabilities and/or their family members, 6 professionals at Madwaleni District Hospital, 7 professional health workers, and 5 unskilled health workers	South Africa
Davidsson & Södergård (10)	Qualitative (Structured interviews)	Nine participants with physical disabilities, aged 46–87 years (five women and four men)	United States (Rural Louisiana)

**Table 2:** Characteristics of the included studies, detailing healthcare access and utilization, barriers to access and key findings.

Study	Healthcare access and utilization	Barriers to access	Key findings
Moscoso-Porras et al. (3)	In rural areas, only 43.6% of people with disabilities (PWD) reported the existence of a rehabilitation center compared to nearly 100% in urban areas. Health center utilization was 71.7% in rural areas vs. 87.5% in urban areas, while rehabilitation center utilization was significantly lower at 10% in rural areas compared to 27.8% in urban areas.	Architectural barriers were significantly more prevalent in rural areas, with common issues including the absence of ramps, handrails, elevators, and adapted bathrooms. These physical and environmental barriers greatly reduced the likelihood of people with disabilities utilizing rehabilitation centers.	The study highlights significant disparities in access to healthcare and rehabilitation services between rural and urban areas, with rural regions having underserved healthcare infrastructure that results in lower utilization rates and poorer health outcomes for people with disabilities. It emphasizes the urgent need for policy interventions to improve healthcare infrastructure in rural areas.
Zhao and Wang (6)	In China, the prevalence of unmet needs among people with disabilities includes medical (15.4%), care (10.2%), rehabilitation (45.6%), and accessibility (13.7%). Rural residents and individuals with multiple disabilities are disproportionately affected.	Rural residents faced significant barriers to healthcare access, including lower income, higher costs, lack of transportation, and fewer healthcare providers, compared to urban residents who reported fewer issues with accessibility and affordability. Rural hukou status was associated with a 13%–40% increase in unmet needs, driven by community-level factors such as limited access to health facilities, low social participation, and insufficient health professionals.	Rural residents and individuals with multiple disabilities face higher unmet healthcare needs, with physical disabilities having the greatest unmet rehabilitation needs. Barriers include economic constraints, transportation issues, and lack of services. Community-level improvements, such as rehabilitation stations and social activities, alongside targeted policies to enhance infrastructure, financial support, and transportation, are essential to address these disparities and improve equity.
Gimm and Ipsen (7)	Rural adults with disabilities perceive a lower need for healthcare services compared to their urban counterparts and are less likely to access mental health services and preventive care.	Transportation issues, fewer healthcare providers, and greater distances to services were significant barriers in rural areas. Additionally, cost and lack of insurance were more pronounced barriers in rural regions.	The study highlights the need for tailored healthcare services in rural areas to address unmet needs and emphasizes the importance of policy changes to reduce barriers and improve access for rural residents.
Pinto and Muhache (2)	Rural areas face poor availability of specialized health services, with low utilization of available services due to both perceived and real barriers. Additionally, rural areas have fewer healthcare facilities compared to urban areas.	Rural areas face greater healthcare barriers due to fewer facilities and more significant transportation challenges. Key barriers include physical barriers such as a lack of ramps and inaccessible transportation, economic barriers like high service costs, and social barriers such as stigma and discrimination.	The study identifies significant barriers to accessing health services for people with disabilities and highlights the need for policy and infrastructural changes to improve access.
Zhang et al. (4)	Inadequate access to healthcare associated with higher odds of IADL and ADL disability, cognitive impairment, and all-cause mortality. Stronger associations in rural areas.	Higher inadequate access to healthcare in rural areas (9.1%) compared to urban areas (5.4%).	Higher odds of IADL and ADL disability, cognitive impairment, and all-cause mortality in rural areas. Inadequate access associated with 33%–37% increased mortality in urban areas, 28%–29% in rural areas.
Hamilton et al. (8)	Limited availability of primary care physicians and specialists, particularly in rural areas. Participants used urgent care clinics due to lack of appointments.	Major barriers included transportation issues, insurance-related challenges, inadequate patient-centered care, and communication difficulties with healthcare providers.	Rural residents faced greater challenges, including transportation difficulties and fewer available healthcare providers, often leading to delays in receiving care. Urban residents had easier access to services.
Grut et al. (9)	The study explored healthcare access in South Africa's rural Amathole district, where services included primary healthcare clinics, community-based initiatives, and a rehabilitation team. Despite these services, utilization was limited by systemic and contextual challenges, such as insufficient outreach and reliance on monthly professional visits.	Barriers included poor infrastructure, financial constraints, cultural beliefs, and systemic issues like professional shortages and bureaucratic hurdles. These factors made accessing healthcare arduous, particularly for individuals with disabilities living in remote areas.	Healthcare access for people with disabilities in resource-poor settings is hindered by interconnected social, cultural, and systemic barriers. The study emphasized the need for community-centered approaches, better infrastructure, and policies addressing poverty and disability to improve healthcare equity and utilization.
Davidsson & Södergård (10)	Participants were all dependent on transportation to access their primary and specialized doctors, and they often had one or more local primary care doctors and at least one specialized doctor out of town. Most interviewees were satisfied with the healthcare they received and felt they got the majority of needed care, especially if they were on Medicare/Medicaid.	Rural residents reported more struggles accessing health and rehabilitation centers.	The ability to access healthcare and the feeling of receiving needed care were closely linked to insurance coverage. The main facilitators to access were social support (primarily from family and friends), transportation assistance, continuity of care, and trust in doctors. People with disabilities in rural areas face multiple barriers but also have facilitators that help them overcome these obstacles.

**Study selection**: The PRISMA flow diagram provides a visual summary of the study selection process.

### Barriers to healthcare access in rural areas

Access to healthcare for disabled individuals in rural areas is shaped by a complex interplay of environmental, personal, and socioeconomic factors. A primary obstacle is the lack of physical and healthcare infrastructure. Rural areas often have fewer healthcare facilities and a limited number of healthcare professionals, leading to restricted access to specialized medical care, state-of-the-art clinics, and rehabilitation centers (3). This scarcity contributes to higher rates of unmet medical and rehabilitation needs, with rural residents having 13%–40% higher unmet healthcare needs than their urban counterparts. Individuals with more than one disability or those with physical disabilities are particularly affected, reporting the highest rates of unmet needs for care and rehabilitation services. These disparities are further compounded by a shortage of supportive infrastructure like transport networks (6).

Transportation presents a significant, specific barrier (Table 2). Public transportation networks in rural areas are often weak or nonexistent, and specialized transport for people with assistive devices is scarce. Long distances, poor roads, and the high cost of private transport can make independent travel impossible, deterring access to care, especially for those with chronic diseases or multiple disabilities. These barriers can lead to delayed diagnoses and poorer health outcomes. Financial constraints, such as low incomes and inadequate insurance coverage for transport, further reinforce these difficulties, placing rural residents with disabilities at a significant disadvantage (3).

In rural Louisiana, these issues are particularly pronounced, with people with disabilities facing a distinct set of challenges and support systems. Participants in one study cited a lack of transportation, limited local specialized care, and inadequate insurance coverage as primary barriers. Financial limitations were a significant concern, as many were unable to work due to their disabilities, leading to high out-of-pocket spending on medications and appointments. For some, this meant they couldn't afford all their necessary medications or even avoided doctor visits because of the cost. The study identified an especially vulnerable group as those with incomes too high for Medicare/Medicaid eligibility who still struggled to pay for healthcare, feeling they “fall through the cracks” of the system. Despite these obstacles, key facilitators were also present, including strong social support from family and friends, transportation assistance, continuity of care, and a high level of trust in their physicians, which helped them navigate these challenges (10).

In addition to these physical barriers, architectural inaccessibility within healthcare facilities remains a major impediment. The lack of ramps, handrails, lifts, and accessible toilet facilities is a widespread issue in medical centers, a problem exacerbated in rural settings where such accommodations are less common. This averseness towards using rehabilitation units points to a need for improved infrastructure that adheres to accessibility standards (3). Beyond physical access, communication and literacy barriers also hinder the delivery of health information, necessitating the development of special materials like Braille, large-print, audio formats, and digital media to improve accessibility (11).

Socioeconomic and cultural factors also complicate access (9). Individuals with disabilities in rural regions have higher unmet needs due to lower educational attainment, fewer job opportunities, and financial limitations that often prevent them from affording necessary services. Cultural attitudes may also contribute to a reluctance to seek care, especially for preventive services (5). Research shows that behavioral norms vary significantly between rural and urban populations, with rural residents having lower rates of seat belt use, higher smoking rates, and lower vaccination rates (12). Additionally, studies have found that rural residents are more likely to report no need for preventive care like dental and mental health counseling. For example, a logistic regression analysis showed that rural residents were more than twice as likely to report no need for mental health counseling compared to their urban counterparts, even when controlling for other factors (7). These findings suggest that addressing deeply rooted behavioral norms and perceived needs is crucial for making preventive care services effective, even when access is improved.

### Barriers to healthcare access in urban areas

Urban areas, despite their abundance of healthcare facilities, present a unique set of challenges for people with disabilities. A primary obstacle is system overcrowding, which can lead to frustratingly long wait times and potentially compromise the quality of care. This is particularly difficult for individuals with complex and ongoing health needs who require timely access to services. Additionally, socioeconomic disparities in cities create significant barriers. Residents in low-income urban areas often face a lack of insurance and limited finances, which restricts their access to high-quality facilities and can exacerbate healthcare inequalities (6).

Another major hurdle, common to both urban and rural settings, is navigating health insurance (8). Even with a wider selection of providers in cities, many people struggle to understand the specifics of their plans, like who is in-network, what services are covered, and what their out-of-pocket costs will be. This administrative complexity can lead to delayed treatment or even prevent it altogether due to communication breakdowns between providers and insurance companies. This issue is often more severe for rural residents, who have fewer local in-network options and may have to travel long distances or pay out-of-pocket for necessary services.

The bureaucratic burden of securing care is a significant concern for people in both locations. The time and effort required to get pre-authorizations for treatment, along with the frequent rejection of claims for essential procedures, are major stressors. This burden disproportionately affects disabled individuals who need consistent, long-term care. A lack of transparent communication between insurers and physicians only adds to this complexity. While urban residents might have more access to local support programs to help with these hurdles, rural residents often don't have this same assistance.

Finally, physical and architectural barriers persist even in urban environments. Despite the concentration of facilities, a lack of basic accessible features like ramps, handrails, elevators, and accessible restrooms can still make it difficult for people with disabilities to get the care they need (3). This highlights that the mere presence of numerous healthcare facilities in a city doesn't guarantee equitable access for all residents (See Table 2).

### Facilitators to healthcare access in rural areas

Community-based interventions are vital for improving healthcare access for people with disabilities in rural communities. These programs provide essential services like transportation and home healthcare, often through community-based health workers. Research shows that communities with resources such as rehabilitation facilities and social programs have fewer unmet needs among their disabled residents. However, this isn't a complete solution. For example, in China, even after accounting for these community factors, rural residency was still a predictor of unmet health needs, suggesting that other barriers persist (6). This means that while community-based interventions are critical, they must be part of a larger strategy to fully address the healthcare challenges faced by disabled individuals in rural areas.

Mobile health units also play a key role in bringing healthcare directly to rural communities (13). These units eliminate the need for long and difficult travel to distant medical centers, which is a major advantage for people with limited mobility. Mobile clinics are often equipped with advanced medical equipment and staffed by professionals who can offer a wide range of services, from general check-ups to specialized care like rehabilitation. By bringing flexible and accessible services directly to communities with limited resources, these units are highly effective in reducing healthcare disparities and improving health outcomes for rural populations with disabilities.

### Facilitators to healthcare access in urban areas

Urban areas, with their well-developed infrastructure, offer unique facilitators for healthcare access. One of the most significant is telemedicine and other technological advances. In cities, where internet connectivity and computer literacy are higher, telemedicine is a viable alternative for people with physical barriers to visiting health centers (14). For those with mobility impairments, it eliminates the need for travel, which is a huge benefit for patients who need regular check-ups or special care, as it minimizes the physical and logistical challenges of navigating a busy city (6). Telemedicine also helps address the issue of overcrowding and long wait times by allowing patients to schedule visits faster than through traditional means.

Beyond telemedicine, urban areas benefit from other technological innovations like electronic health records (EHR) and AI-powered platforms (15). These systems help improve coordination among specialists and primary care providers, which is essential for people with disabilities who often need interdisciplinary treatment. AI can assist with early illness detection and provide predictive recommendations. Together, these technologies create a more integrated and responsive healthcare system, giving disabled individuals greater autonomy and control over their health without requiring repeated physical visits.

Finally, urban areas are able to offer a greater concentration of specialized healthcare services and professionals (11). For people with disabilities who require specific and complex interventions, this is a significant advantage. For example, studies show that while only about 44% of rural residents had access to a rehabilitation center, nearly all urban residents reported the same availability (3). This higher concentration of professionals also offers a broader choice of care, which can help reduce wait times for necessary services. Furthermore, well-developed urban transportation systems make it easier for people with disabilities to reach these facilities.

## Discussion

This systematic review reveals a profound divide in healthcare access for people with disabilities, a divide shaped by their rural or urban environment. While rural residents face a complex web of challenges, including inadequate physical infrastructure, difficult transportation, and socioeconomic inequality, urban residents contend with their own set of barriers, primarily system overcrowding, extended wait times, and administrative complexities with insurance. This fundamental distinction makes it clear that a universal healthcare strategy is insufficient. A more effective approach demands flexible, tailored interventions that directly address the specific obstacles in each setting.

One of the most significant and pervasive barriers for rural residents with disabilities is the lack of physical and healthcare infrastructure. Our review consistently found that inadequate transportation and inaccessible facilities often prevent people from even attempting to seek care (7). This is especially difficult for those with multiple or chronic disabilities, who are frequently forced to delay or forgo essential medical appointments and rehabilitation services, leading to poorer health outcomes. The shortage of healthcare providers in these areas exacerbates the problem, leaving rural residents with less access to both basic and specialized care. This trend of inadequate access and its association with worse health outcomes has been consistently observed in studies from countries like China, the U.S., and many low- and middle-income countries (6).

It is important to acknowledge that the term “disability” encompasses a broad spectrum of conditions, including physical, intellectual, cognitive, and mental impairments**.** The studies included in this review varied in how they defined and classified disability, with most focusing primarily on physical limitations and fewer examining cognitive or psychosocial aspects. Consequently, our synthesis treats disability as a general category due to limited data disaggregation in the source literature. This represents an inherent limitation, as the barriers and facilitators to healthcare access can differ substantially across disability types. For example, individuals with mobility impairments often encounter architectural and transportation barriers, whereas those with intellectual or cognitive disabilities are more affected by communication challenges, stigma, and provider bias. These distinctions highlight the need for future research to adopt a more differentiated approach that examines disability-specific access barriers and context-specific facilitators.

Earlier evidence supports the compounded nature of barriers experienced by people with disabilities in rural settings (16). reviewed 86 studies and found that rural residents with disabilities face unique structural and systemic challenges, including geographic isolation, inadequate healthcare infrastructure, and a shortage of trained professionals capable of addressing disability-related health needs. Local healthcare systems often rely on paraprofessionals, outreach programs, and mobile units to fill service gaps, but these approaches remain limited in scope and sustainability. The authors emphasized the importance of developing and testing alternative delivery models, such as regional centers of specialized care and telemedicine consultations between urban specialists and rural practitioners, to improve access and continuity of care. Their findings underscore that many of the barriers identified nearly three decades ago persist today, highlighting the urgent need for innovative, evidence-based strategies tailored to rural populations with disabilities.

Urban areas, while offering a greater concentration of healthcare resources, are not without their own flaws. Despite a higher number of hospitals and specialists, overcrowding and long wait times remain major issues. Studies show that even with more frequent contact with healthcare providers, urban residents with disabilities still struggle to access timely care (5). This often forces them to rely on urgent care clinics, which can lead to fragmented care and, ultimately, worse health outcomes. Furthermore, the administrative complexity of health insurance is a significant barrier across the board. Many people struggle to understand their plans, navigate pre-authorization processes, and afford out-of-pocket costs, a burden that is particularly heavy for those with ongoing healthcare needs (8). This issue can be more severe for rural residents, who have fewer local in-network options and may need to travel long distances or pay out-of-pocket for essential services.

To tackle these persistent problems, our review points to a clear need for a multi-faceted approach. In rural areas, we should focus on community-based interventions, such as mobile health clinics and telehealth, to overcome geographical challenges. For telehealth to be truly effective, though, there needs to be greater investment in digital infrastructure and training for both providers and patients. We also need to repair and improve rural healthcare infrastructure and ensure all facilities meet accessibility standards (3). In cities, policies should aim to reduce system overcrowding and simplify the administrative side of healthcare. Expanding insurance coverage and making facilities more physically accessible with features like ramps and elevators are also critical steps.

Recent evidence further supports the need for collaborative and multi-sector approaches to address healthcare accessibility for individuals with disabilities (17). Conducted a systematic review examining accessibility barriers and found that despite policy efforts such as the Americans with Disabilities Act, many healthcare facilities remain ill-equipped to meet the needs of disabled patients. Their analysis identified recurring issues, including inadequate physical accommodations, poor communication support, and limited provider training on disability care. Importantly, they propose a Collaborative Partnership Model that integrates academic institutions, advocacy organizations, and healthcare education providers to promote inclusive practices through joint training, community engagement, and systemic reform. This model aligns closely with the findings of our review, reinforcing that sustainable improvement in healthcare access requires coordinated action across policy, education, and service delivery sectors.

Although several facilitators were identified in both rural and urban contexts, their mechanisms of impact differ considerably. In rural settings, facilitators such as telemedicine, mobile health units, and community-based programs primarily address structural barriers related to distance, provider shortages, and transportation challenges. In contrast, in urban areas, the same technologies function to enhance efficiency by reducing waiting times and improving care coordination within dense and complex healthcare systems. This distinction suggests that facilitators are not universally applicable but context-dependent, with their effectiveness shaped by local infrastructure, population density, and resource availability. Integrating these insights highlights the importance of designing adaptive, location-specific strategies, where rural initiatives prioritize outreach and accessibility, and urban systems focus on technological integration and service optimization.

Ultimately, achieving equitable healthcare for people with disabilities means moving beyond a single solution and creating specific strategies that address the unique barriers of each setting. We must recognize that the challenges in a small rural town are fundamentally different from those in a large, bustling city, and our policies need to reflect that. Only by addressing these complex, context-specific barriers can we work toward a more equitable healthcare system for all.

## Implications for policy and practice

The findings from this systematic review have important takeaways for policymakers, healthcare providers, and community organizations who want to reduce healthcare inequities for people with disabilities. To truly bridge these gaps, we need a response that's as unique as the challenges themselves, with interventions specifically tailored for both rural and urban dwellers (6).

### Policy makers should give high priority to the allocation of financial resources and funding aimed at eliminating inequities in access to healthcare services

Policymakers need to make it a priority to allocate money and resources to eliminate inequities in healthcare access. This means providing funding for telemedicine centers and offering financial assistance to low-income individuals with disabilities, especially in rural communities. Hospitals should also be required to meet accessibility standards, creating a genuinely inclusive healthcare system (3). Shifting resources to help disadvantaged groups in both rural and urban areas is a critical step toward reducing the disparity in healthcare access.

### Engagement with the local stakeholders and communities is crucial for the sustainability of healthcare interventions

Engaging with local communities and stakeholders is the key to making healthcare interventions successful in the long run. Community-based programs, which are built on local knowledge and resources, can be incredibly effective in helping people get the care they need in places where barriers are high. For example, in rural areas, programs that encourage social engagement and community-based rehabilitation show great promise. Involving local organizations in designing and implementing these interventions ensures the solutions are relevant to the community and are more likely to last.

### There are considerable unmet healthcare needs among PWD, especially in rural regions

There are significant unmet healthcare needs among people with disabilities, and it's especially apparent in rural areas. Nearly 45.6% of adults with disabilities report they need but don't receive rehabilitation services, 15.4% need medical care, 13.7% need more accessible facilities, and 10.2% need more care services. These disparities are particularly pronounced in rural regions, where people with disabilities are 13%–40% more likely to have unmet healthcare needs than their urban counterparts (6). To tackle these disparities, policy interventions must improve accessibility to health facilities, create more opportunities for social participation, and increase the number of health workers in rural regions.

### Further studies need to investigate the underlying determinants of healthcare access disparities among PWD

More studies are needed to investigate the root causes of healthcare access disparities among people with disabilities. Researchers should explore how stigma, social beliefs, and cultural values influence healthcare access, especially in rural and impoverished communities. We also need more long-term studies to determine how effective telemedicine and community interventions are at removing healthcare barriers for people with disabilities in different geographical settings.

### There is a need to improve the training of health providers so that they are able to cater to the unique needs of PWD

Healthcare providers need better training so they can effectively care for the unique needs of people with disabilities. This training should make providers more aware of the challenges people with disabilities face and the various stigmas that hinder their access to healthcare. The goal is to provide a more inclusive environment where individuals with disabilities can get the services they need without facing discrimination.

### Lastly, this systematic review calls for specific policy responses and community-level interventions that take into account the specific needs of PWD living in rural and urban settings

Finally, this systematic review calls for specific policy responses and community-level interventions that are designed with the particular needs of people with disabilities in both rural and urban areas in mind. Policymakers must invest in transport infrastructure, telemedicine, and rehabilitation centers, while also guaranteeing accessible healthcare facilities. Only by addressing these complex, multi-faceted problems can we work toward a truly equitable healthcare system for all.

## Strengths and limitations

This systematic review has several key strengths that bolster the credibility of its findings. Our methodology was built on a comprehensive search strategy across multiple databases, which increases our confidence that we captured the vast majority of relevant published literature on this topic. The inclusion criteria were robust and well-defined, ensuring that the studies we analyzed were directly relevant to our research question and met a certain standard of quality. Furthermore, the use of standardized tools for data extraction and quality assessment, such as the Mixed Methods Appraisal Tool (MMAT) and the PRISMA statement, helped to ensure consistency and minimize the risk of reviewer bias throughout the process.

However, a few limitations must be acknowledged when interpreting our findings. A primary challenge was the heterogeneity of the included studies. The research we synthesized came from different countries, used varied methodologies (both quantitative and qualitative), and sometimes applied different definitions of disability or healthcare access. This made it difficult to make direct comparisons and generalize the findings across all populations and settings. Additionally, there is always the possibility of publication bias, where studies with significant or “positive” results are more likely to be published than those with null findings. Our decision to exclude non-English language studies also represents a limitation, as we may have unintentionally missed important research from other parts of the world. Finally, by relying solely on peer-reviewed literature, we may have overlooked valuable insights contained in grey literature, such as government reports, conference proceedings, or unpublished research that could have provided a more complete picture of the issue. These limitations should be considered when applying the results of this review to broader contexts.

## Conclusion

This systematic review underscores the significant disparities in healthcare access faced by individuals with disabilities, with rural areas exhibiting a greater prevalence of unmet needs compared to urban settings. The synthesis of evidence highlights that structural barriers, including limited healthcare infrastructure, transportation challenges, and socioeconomic disadvantages, disproportionately impact rural populations, particularly those with multiple disabilities. Addressing these fundamental inequities is imperative for the realization of equitable healthcare access for all.

Bridging the rural-urban healthcare divide necessitates a multifaceted approach. The development of robust infrastructure, the strategic expansion of healthcare services, and the implementation of policies that prioritize accessibility are critical components for reducing these disparities. Furthermore, culturally sensitive interventions and public health strategies must be employed to mitigate stigma, improve health literacy, and promote the utilization of preventive and specialized care.

Policymakers are urged to consider both geographic disparities and the unique needs associated with various disabilities when designing and optimizing healthcare systems. Tailored solutions, such as the strategic deployment of telehealth and mobile clinics, can play a pivotal role in improving access in underserved areas. By directly addressing the underlying socioeconomic and structural determinants of health, healthcare systems can be transformed to become more inclusive and responsive to the diverse needs of people with disabilities.

In conclusion, achieving equitable healthcare access for individuals with disabilities requires a comprehensive and sustained effort to close the rural-urban gap. The prioritization of inclusive healthcare policies, the enhancement of infrastructure, and the mitigation of key barriers are critical steps toward ensuring that all individuals, irrespective of their location or disability, can receive the care they need.
