# Correction: Bridging healthcare disparities: a systematic review of healthcare access for disabled individuals in rural and urban areas

**DOI:** 10.3389/frhs.2026.1785070

**Published:** 2026-01-22

**Authors:** Amer Mesmar, Godfrey Mbaabu Limungi, Mohammed Elmadani, Klara Simon, Osama Hamad, Livia Tóth, Eva Horvath, Orsolya Mate

**Affiliations:** 1 Doctoral School of Health Sciences, Faculty of Health Sciences, University of Pécs, Pécs, Hungary; 2 Faculty of Dentistry, University of Jordan, Amman, Jordan; 3 Institute of Emergency Care, Pedagogy of Health and Nursing Sciences, Faculty of Health Sciences, University of Pécs, Pécs, Hungary

**Keywords:** healthcare access, disability, health disparities, rural health, urban health, telemedicine, healthcare barriers, equity in healthcare

**Author affiliation**

Affiliation Doctoral School of Health Sciences, Faculty of Health Sciences, University of Pécs, Pécs, Hungary was erroneously assigned to author Orsolya Mate. The correct affiliation for Orsolya Mate should be Institute of Emergency Care, Pedagogy of Health and Nursing Sciences, Faculty of Health Sciences, University of Pécs, Pécs, Hungary.

